# Analysis of two-year hearing outcomes and risk factors in newborns in the province with the highest annual birth rate in Türkiye

**DOI:** 10.1038/s41598-026-52383-0

**Published:** 2026-05-07

**Authors:** Servet Erdemes, Müslüm Ayral, Tahir Nazlıoğlu, Şule Öztürk

**Affiliations:** 1 Faculty of Medicine, Department of Otorhinolaryngology, Head and Neck Surgery, Harran University, Şanlıurfa, Turkey; 2 Department of Otorhinolaryngology, Head and Neck Surgery, Gazi Yaşargil Training and Research Hospital, Diyarbakır, 21000 Turkey; 3 Faculty of Medicine, Department of Audiology, Harran University, Şanlıurfa, Turkey; 4 Department of Audiology, Sanliurfa Training and Research Hospital, Şanlıurfa, 63200 Turkey

**Keywords:** Hearing loss, Newborn, Risk factors, Diseases, Health care, Medical research, Risk factors

## Abstract

This study aimed to evaluate risk factors for neonatal hearing loss among newborns referred to tertiary audiology centers and to investigate their association with confirmed hearing outcomes. A total of 1,869 newborns referred to tertiary audiology centers after failed newborn hearing screening or due to established risk factors between January 2021 and January 2023 were retrospectively evaluated using the OtoAccess database. The analysis was restricted to referred newborns rather than the entire birth cohort. During 2021–2022, a total of 113,665 births were recorded in Şanlıurfa, of which 1,869 newborns were referred for diagnostic evaluation at tertiary audiology centers. Hearing loss was detected in 8.4% of infants. Among newborns with at least one documented risk factor, 48 (3.5%) met the clinical criteria for cochlear implantation. Family history of hearing loss and syndromic disorders were significantly associated with neonatal hearing loss (*p* < 0.05). No statistically significant differences were observed between infants with and without hearing loss in terms of mechanical ventilation, ototoxic drug exposure, hyperbilirubinemia, neurodegenerative disease, TORCH infections, cerebral pathology, or maternal diseases. Nearly half of affected infants had severe-to-profound hearing loss, highlighting the importance of early cochlear implant evaluation in referred neonatal populations. Family history of hearing loss and syndromic disorders were identified as significant factors associated with neonatal hearing loss in this referral-based cohort. The high prevalence of consanguineous marriage in the region may be associated with the burden of familial hearing loss. Additionally, the occurrence of hearing loss in infants without identifiable risk factors underscores the importance of universal newborn hearing screening and timely referral to specialized audiology centers.

## Introduction

Hearing is essential for the normal development of speech, cognition, and social interaction in newborns. Previous studies have shown that untreated hearing loss (HL) in early life negatively affects language acquisition, intellectual development, and psychosocial outcomes^1,2^. The global prevalence of congenital HL is estimated to range between 0.5 and 5 per 1,000 live births. Early detection through neonatal screening and intervention before six months of age significantly improves long-term developmental outcomes^3–5^.

Universal newborn hearing screening programs follow standardized international guidelines and commonly employ otoacoustic emission (OAE) and/or auditory brainstem response (ABR) testing. In Türkiye, newborn hearing screening became a nationwide public health program in 2012 and is currently implemented across all provinces. Risk factors for neonatal HL have been defined by the Joint Committee on Infant Hearing (JCIH)^6,7^.

Şanlıurfa, the province in which this study was conducted, had the highest birth rate in Türkiye in 2021 and 2022 and is also characterized by a high prevalence of consanguineous marriages, both of which may influence the epidemiology of neonatal HL^8–10^. The region’s total fertility rate substantially exceeds the national average, and demographic factors such as migration and traditional perinatal practices may further affect screening follow-up and early intervention processes^11–13^. These characteristics make Şanlıurfa a particularly informative setting for evaluating regional determinants of neonatal hearing outcomes and for guiding public health planning.

This study aimed to evaluate risk factors associated with HL and to determine their relationship with confirmed hearing outcomes in newborns referred to tertiary audiology centers in this high-fertility region.

Unlike prior studies that primarily report population-level screening prevalence, this study investigates two-year longitudinal hearing outcomes, cochlear implant candidacy, and regional genetic–demographic characteristics within a large referral-based neonatal cohort. This approach provides clinically relevant evidence for targeted follow-up strategies and genetic counseling policies in regions with elevated birth rates and consanguinity.

## Materials and methods

### Study design and population

Newborns who failed newborn hearing screening and/or were referred to tertiary audiology centers at Harran University Faculty of Medicine Hospital and Şanlıurfa Training and Research Hospital due to established risk factors between January 2021 and January 2023 were included in this retrospective study. Medical records were reviewed using the OtoAccess database.

Ethical approval was obtained from the Clinical Research Ethics Committee of Harran University Faculty of Medicine (HRÜ/24.02.49), and the study was conducted in accordance with the Declaration of Helsinki. Clinical, demographic, audiological, and risk-factor data were extracted from electronic medical records using a predefined data collection framework. Only routinely recorded clinical data were used, without any direct patient contact or additional data collection.

The timing of confirmatory diagnostic ABR evaluation varied depending on referral patterns. The median age at diagnostic ABR was 6 weeks (range: 2–12 weeks). Therefore, the study cohort consisted of referred newborns rather than the entire regional birth population.

### ENT examination and audiological assessment

All infants underwent ENT examination at both centers prior to ABR testing. The examination included otoscopic evaluation of the external auditory canal and tympanic membrane, assessment of middle ear effusion and congenital auricular anomalies, and inspection of the nasal cavity and oropharynx for airway patency and syndromic features. Additional endoscopic or imaging evaluations were performed only when clinically indicated.

Infants diagnosed with middle ear effusion received appropriate treatment, followed by reassessment after resolution. Tympanometry was performed when middle ear pathology was suspected.

All infants who completed ENT evaluation underwent comprehensive diagnostic auditory brainstem response (ABR) testing. Clinical data and medical histories were evaluated according to the Joint Committee on Infant Hearing (JCIH) risk-factor framework^7^, including NICU admission, mechanical ventilation, ototoxic medication exposure, hyperbilirubinemia, family history of hearing loss, syndromic disorders, neurodegenerative diseases, TORCH infections, cerebral pathologies, and maternal illnesses. Each factor was analyzed individually for its association with hearing loss occurrence and severity.

Parental consanguinity data were not consistently available in the retrospective records and were therefore not included in the analysis. Risk factor comparisons were performed only among infants with at least one documented risk factor (*n* = 1369).

### Auditory brainstem response (ABR) testing

Diagnostic ABR testing was performed using the Interacoustics Eclipse EP25 system. ABR measures electrical potentials generated by auditory stimuli along the auditory pathway to the brainstem via surface scalp electrodes^14^. All assessments were conducted by a certified audiologist while infants were in a natural sleep state, thereby minimizing movement artifacts and eliminating the need for sedation.

### Definition of hearing loss

Hearing loss severity was categorized according to standard threshold ranges:

- Mild: 26–40 dB.
- Moderate: 41–55 dB.
- Moderate-severe: 56–70 dB.
- Severe: 71–90 dB.
- Profound: >90 dB.

### Cochlear implant candidacy

In Türkiye, cochlear implantation reimbursement criteria are defined by the Social Security Institution. Cochlear implantation is indicated for patients with bilateral severe-to-profound hearing loss who show no functional benefit after at least three months of hearing aid use^15^.

In this study, infants meeting these national clinical criteria were classified as cochlear implant (CI) candidates. For the analysis presented in Table 1, infants were categorized as “CI candidate (Yes)” if documentation confirmed bilateral severe-to-profound hearing loss with insufficient benefit from hearing aids. Infants were classified as “Not a CI candidate (No)” if they had normal hearing, mild-to-moderate hearing loss, unilateral hearing loss, or incomplete CI candidacy data.

**Table 1:** Distribution of descriptive characteristics.

		n (%)
**ABR Screening Results**	**Bilateral pass**	750 (40,1)
	**Fail**	881 (47,1)
	**No ABR test performed**	238 (12,7)
**HL**	**No HL**	1712 (91,6)
	**HL**	156 (8,4)
**Cochlear Implant Candidacy**	**No**	1791 (95,8)
	**Yes**	78 (4,2)
**Risk Factor**	**None**	500 (26,8)
	**Present**	1369 (73,2)

HL: Hearing Loss.

Among the 156 infants diagnosed with hearing loss, severity distribution was heterogeneous: mild (16.7%, *n* = 26), moderate (20.5%, *n* = 32), moderate-severe (12.8%, *n* = 20), severe (21.8%, *n* = 34), and profound (28.2%, *n* = 44). Severe-to-profound bilateral hearing loss constituted nearly half of all confirmed cases, representing the primary subgroup considered for cochlear implantation (Table 2).

**Table 2:** Severity distribution of hearing loss among infants diagnosed with HL (*n* = 156).

Hearing loss severity	*n*	%
Mild	26	16.7
Moderate	32	20.5
Moderate-severe	20	12.8
Severe	34	21.8
Profound	44	28.2
**Total**	**156**	**100**

### Two-year hearing outcomes and follow-up procedures

#### Definition of two-year hearing outcomes

This retrospective cohort study evaluated hearing outcomes during the second year following the initial referral. Follow-up data were obtained from the OtoAccess database, which records routine clinical assessments after the initial evaluation. Audiological assessments were conducted during scheduled follow-up visits and included diagnostic ABR testing when indicated, otoscopic examination, tympanometry, and age-appropriate behavioral assessments. Visual reinforcement audiometry (VRA) was used for infants and young toddlers when feasible, while conditioned play audiometry (CPA) was applied in children approaching 24 months with appropriate developmental abilities. Two-year outcomes were categorized as either normal hearing or hearing loss, with severity classified according to dB thresholds (mild to profound). When available, data on laterality (unilateral/bilateral) and suspected type of hearing loss (conductive vs. sensorineural/mixed) were also recorded.

#### Follow-up visit frequency

Follow-up frequency varied according to standard clinical practice and individual patient needs. The number of visits differed among patients depending on clinical progression and adherence to scheduled appointments. Missed follow-up visits were recorded as “loss to follow-up.”

### Statistical analysis

All statistical analyses were performed using SPSS version 27 (Statistical Package for the Social Sciences). Descriptive statistics were used to summarize qualitative and quantitative data. Fisher’s exact test and Pearson’s chi-square test were applied for comparisons of categorical variables. Statistical significance was set at *p* < 0.05 with a 95% confidence interval. A multivariable logistic regression analysis was also performed to evaluate independent associations between hearing loss and selected variables, including NICU admission, family history of hearing loss, and syndromic disorders.

## Results

### Study population and referral flow

Newborns referred to tertiary audiology centers in Şanlıurfa between January 2021 and January 2023 were included in the analysis. During 2021–2022, a total of 113,665 births were recorded in the region. Among these, 1,869 infants (947 females, 922 males) were referred to tertiary audiology centers, corresponding to a referral rate of 1.64%.

**Fig. 1:**
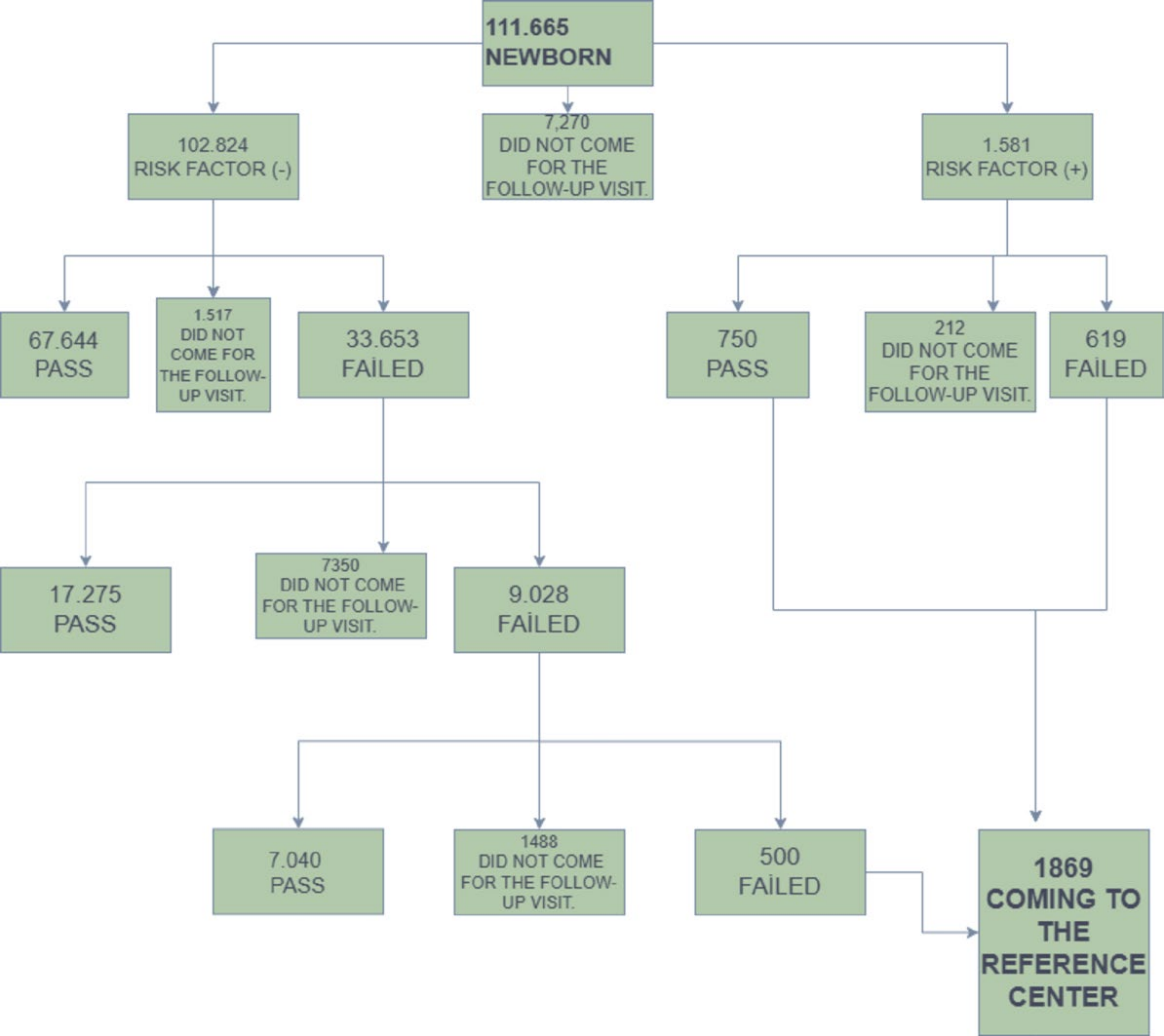
Screening data for all newborns.

**Fig. 2:**
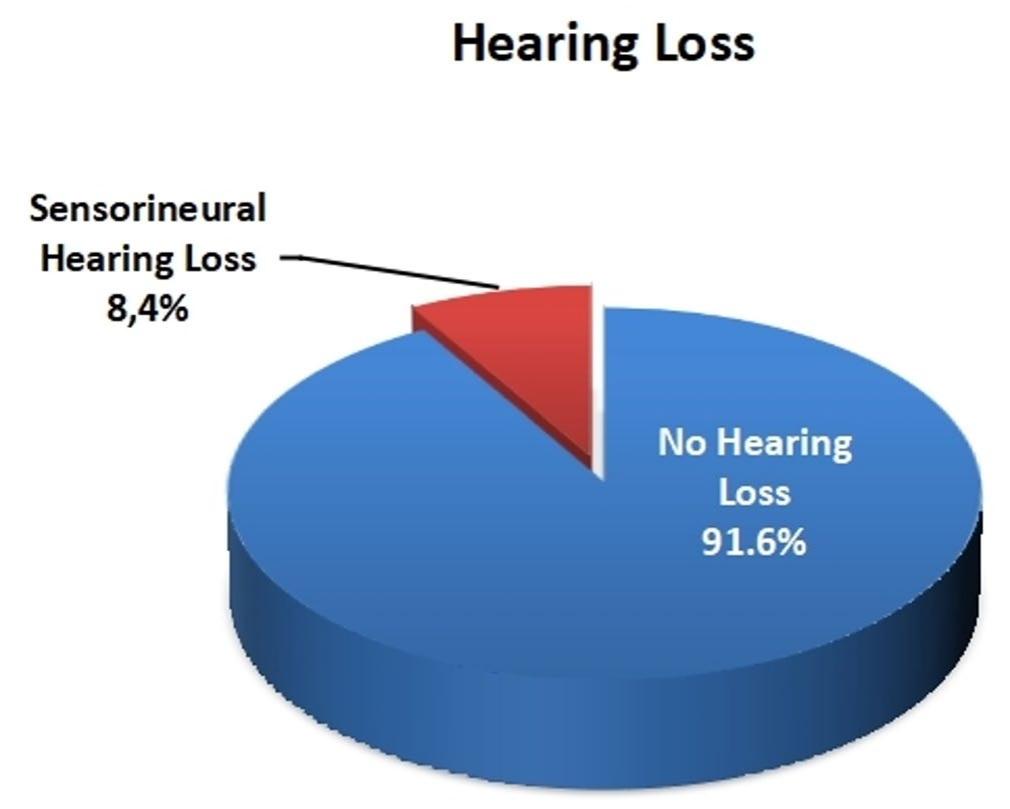
Distribution of hearing loss.

Regional newborn hearing screening outcomes for the entire birth cohort are summarized in Fig. 1. The present analysis focuses specifically on the 1,869 infants referred for diagnostic evaluation after screening failure or due to established risk factors. The results of the diagnostic evaluation are shown in Fig 2. 

### Hearing loss and cochlear implant candidacy

Among the referred infants, 40.1% (*n* = 750) passed ABR screening bilaterally, while 59.9% (*n* = 1,119) failed in one or both ears. Hearing loss was detected in 8.4% (*n* = 156) of cases.

Among infants with at least one documented risk factor (*n* = 1,369), 48 (3.5%) met the criteria for cochlear implantation according to national clinical guidelines, whereas 1,321 (96.5%) did not. Additionally, 26.8% (*n* = 500) of infants had no documented risk factors, while 73.2% (*n* = 1,369) had at least one risk factor. Descriptive characteristics of the study population are presented in Table 1.

### Distribution of risk factors

**Fig. 3:**
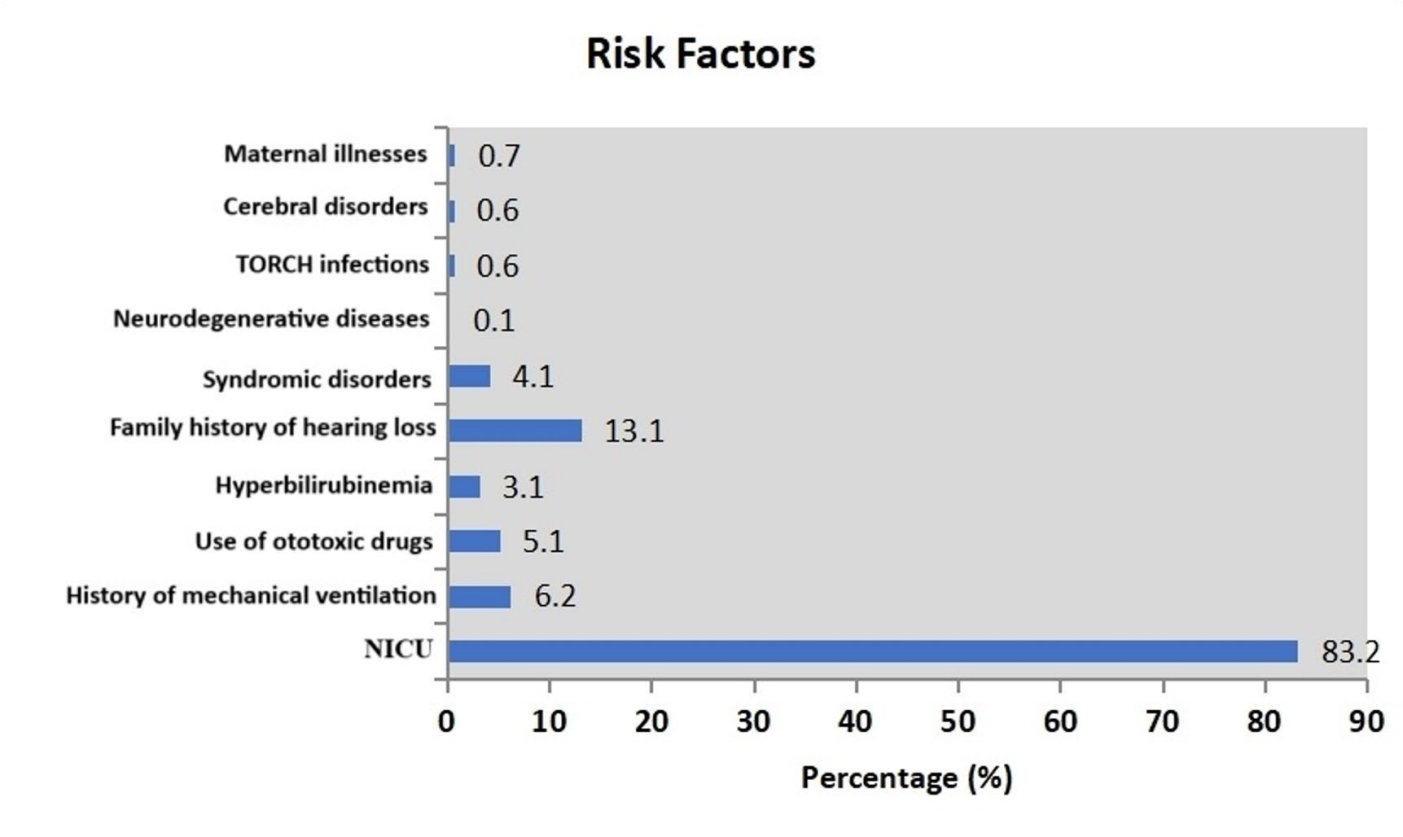
Distribution of risk factors.

The most common risk factor was neonatal intensive care unit (NICU) admission, observed in 83.2% (*n* = 1,139) of infants, followed by a family history of hearing loss in 13.1% (*n* = 180). The distribution of risk factors is shown in Fig. 3.

### Risk factors associated with hearing loss

Syndromic disorders and family history of hearing loss were significantly associated with neonatal hearing loss (*p* < 0.05). No statistically significant differences were observed between infants with and without hearing loss regarding mechanical ventilation, ototoxic drug exposure, hyperbilirubinemia, neurodegenerative diseases, TORCH infections, cerebral disorders, or maternal illnesses (*p* > 0.05).

Among infants with at least one documented risk factor (*n* = 1,369), hearing loss was identified in 87 cases (6.4%). NICU admission was significantly less frequent in infants with hearing loss compared to those without hearing loss (60.9% vs. 84.7%, *p* < 0.001). Detailed comparisons are presented in Table 3.

**Table 3:** Comparison of risk factors according to hearing loss.

Risk factor	Category	No hearing loss (*n* = 1282)	Hearing loss (*n* = 87)	*P* value
**NICU admission**	No	196 (15.3%)	34 (39.1%)	0.001**
	Yes	1086 (84.7%)	53 (60.9%)	
**History of mechanical ventilation**	No	1202 (93.8%)	82 (94.3%)	0.854
	Yes	80 (6.2%)	5 (5.7%)	
**Use of ototoxic drugs**	No	1213 (94.6%)	86 (98.9%)	0.124
	Yes	69 (5.4%)	1 (1.1%)	
**Hyperbilirubinemia**	No	1240 (96.7%)	86 (98.9%)	0.252
	Yes	42 (3.3%)	1 (1.1%)	
**Family history of HL**	No	1134 (88.5%)	55 (63.2%)	0.001**
	Yes	148 (11.5%)	32 (36.8%)	
**Syndromic disorders**	No	1237 (96.5%)	76 (87.4%)	0.001**
	Yes	45 (3.5%)	11 (12.6%)	
**Neurodegenerative diseases**	No	1281 (99.9%)	87 (100.0%)	1.000
	Yes	1 (0.1%)	0 (0.0%)	
**TORCH infections**	No	1274 (99.4%)	87 (100.0%)	1.000
	Yes	8 (0.6%)	0 (0.0%)	
**Cerebral disorders**	No	1274 (99.4%)	87 (100.0%)	1.000
	Yes	8 (0.6%)	0 (0.0%)	
**Maternal illnesses**	No	1273 (99.3%)	87 (100.0%)	1.000
	Yes	9 (0.7%)	0 (0.0%)	

### Risk factors according to cochlear implant candidacy

A family history of hearing loss was significantly more common in cochlear implant candidates compared to non-candidates (*p* < 0.01). Other evaluated risk factors—including mechanical ventilation, ototoxic drug use, hyperbilirubinemia, syndromic disorders, neurodegenerative diseases, TORCH infections, cerebral disorders, and maternal illnesses—were not significantly associated with cochlear implant candidacy (*p* > 0.05).

Among infants with at least one documented risk factor, NICU admission was significantly less frequent in cochlear implant candidates compared to non-candidates (56.3% vs. 84.2%, *p* < 0.001). Detailed comparisons are presented in Table 4.

**Table 4:** Comparison of risk factors according to cochlear implant candidacy among infants with ≥ 1 documented risk factor (*n* = 1369).

Risk factor	No CI (*n* = 1321)	CI candidate (*n* = 48)	*P* value
**NICU admission**			< 0.001
No	209 (15.8%)	21 (43.8%)	
Yes	1112 (84.2%)	27 (56.3%)	
**Mechanical ventilation**			**0.535**
No	1240 (93.9%)	44 (91.7%)	
Yes	81 (6.1%)	4 (8.3%)	
**Ototoxic drug exposure**			**0.511**
No	1252 (94.8%)	47 (97.9%)	
Yes	69 (5.2%)	1 (2.1%)	
**Hyperbilirubinemia**			**0.661**
No	1280 (96.9%)	46 (95.8%)	
Yes	41 (3.1%)	2 (4.2%)	
**Family history of HL**			**< 0.001**
No	1161 (87.9%)	28 (58.3%)	
Yes	160 (12.1%)	20 (41.7%)	
**Syndromic disorder**			**0.442**
No	1268 (96.0%)	45 (93.8%)	
Yes	53 (4.0%)	3 (6.3%)	
**Neurodegenerative disease**			**1.000**
No	1320 (99.9%)	48 (100.0%)	
Yes	1 (0.1%)	0 (0.0%)	
**TORCH infection**			**1.000**
No	1313 (99.4%)	48 (100.0%)	
Yes	8 (0.6%)	0 (0.0%)	
**Cerebral disorder**			**1.000**
No	1313 (99.4%)	48 (100.0%)	
Yes	8 (0.6%)	0 (0.0%)	
**Maternal illness**			**1.000**
No	1312 (99.3%)	48 (100.0%)	
Yes	9 (0.7%)	0 (0.0%)	

## Discussion

The present study provides additional evidence by integrating two-year hearing outcomes, cochlear implant candidacy stratification, and regional genetic–demographic characteristics within a large referral-based neonatal cohort. Few studies from Türkiye have simultaneously evaluated these dimensions, highlighting the clinical relevance of this dataset for targeted follow-up and genetic counseling strategies. The severity distribution demonstrated that nearly half of affected infants had severe-to-profound hearing loss, a proportion higher than that reported in population-based screening cohorts. This finding is consistent with the referral-based nature of the study population and emphasizes the importance of early diagnostic confirmation and timely cochlear implant evaluation in high-risk regional settings. Early diagnosis and treatment of patients from the newborn period is very important in terms of language development, cognitive development and social development^16^. Otherwise, there are negative effects on the child and he/she may lag behind his/her peers in terms of development. The World Health Organization reports that the prevalence of HL in newborns varies from 0.5 to 5 cases per 1,000 live births^17^. In our study, the HL rate was 8.4%, which is higher than in similar studies ^18^,^19^. In contrast to several previous reports, NICU admission was not identified as an independent risk factor for hearing loss. Earlier studies reported increased risk associated with prolonged NICU stay or intensive respiratory support^21,20,27–29^, whereas other investigations found no significant association^22^. The inverse relationship observed in the present cohort is therefore most plausibly explained by referral bias, heterogeneous follow-up pathways, and competing clinical priorities in NICU populations, rather than a protective biological effect. These findings support the view that neonatal hearing-loss risk is multifactorial and context-dependent.

Family history of hearing loss emerged as a strong and significant determinant of both hearing impairment and cochlear implant candidacy, consistent with previous epidemiological observations^23,26,35^. Given the high regional prevalence of consanguinity, this association may indicate an underlying genetic contribution to persistent neonatal hearing loss, in line with studies demonstrating increased autosomal-recessive disorders in consanguineous populations^24,25^. However, parental consanguinity was not systematically recorded in the retrospective dataset, representing an important limitation. Future prospective studies incorporating genetic and familial structure data are required to clarify the contribution of consanguinity to neonatal hearing loss in this population.

Other evaluated risk factors—including mechanical ventilation, ototoxic drug exposure, hyperbilirubinemia, TORCH infections, neurodegenerative disorders, cerebral pathology, and maternal diseases—did not demonstrate statistically significant associations with hearing loss, consistent with several prior reports^32,22,31^. Because some of these conditions were rare in the cohort, the absence of statistical significance may reflect limited statistical power rather than a true lack of effect.

A substantial proportion of infants diagnosed with hearing loss had no identifiable clinical risk factors, paralleling findings from earlier universal-screening studies reporting similar proportions^33, 30,34^. This observation reinforces the essential role of universal newborn hearing screening, even in the absence of recognized risk indicators.

This study has important audiological and methodological limitations. Routine tympanometry and bone-conduction ABR were not available for all infants, preventing definitive differentiation between conductive and sensorineural hearing loss in some cases. Furthermore, the referral-based design limits generalizability to the overall newborn population; therefore, the observed prevalence should be interpreted as a diagnostic rate within a high-risk cohort rather than a population-level estimate. Several evaluated risk factors, including TORCH infections, neurodegenerative disorders, and cerebral pathologies, were rare in this cohort; therefore, the absence of statistically significant associations may reflect limited statistical power rather than a true lack of effect. Additionally, risk-factor comparisons were restricted to infants with documented clinical risk factors, which may limit generalizability to the broader newborn population.

Future prospective, population-based studies incorporating standardized middle-ear assessment, bone-conduction testing, and long-term follow-up are required to determine the true incidence and etiological spectrum of permanent neonatal hearing loss.

From a clinical and public-health perspective, these findings support structured genetic counseling, prioritized longitudinal audiological monitoring for infants with familial or syndromic risk, and standardized early cochlear-implant evaluation protocols in high-fertility regions.

## Conclusion

Family history of hearing loss and syndromic disorders were identified as significant factors associated with neonatal hearing impairment in this referral-based cohort. The detection of hearing loss in infants without identifiable clinical risk factors underscores the importance of universal newborn hearing screening. Additionally, the high proportion of severe-to-profound hearing loss highlights the need for structured follow-up and timely cochlear implant evaluation in high-risk populations.

## Data Availability

The aggregated data presented in this study are available from the corresponding author upon reasonable request.
